# Effects of social deprivation on risk factors for suicidal ideation and suicide attempts in commercially insured US youth and adults

**DOI:** 10.1038/s41598-023-31387-0

**Published:** 2023-03-13

**Authors:** Wenna Xi, Samprit Banerjee, Mark Olfson, George S. Alexopoulos, Yunyu Xiao, Jyotishman Pathak

**Affiliations:** 1 Department of Population Health Sciences, Weill Cornell Medicine, New York, NY 10065 USA; 2 Department of Psychiatry, New York State Psychiatric Institute, Columbia University Irving Medical Center, New York, NY 10032 USA; 3 Department of Psychiatry, Weill Cornell Medicine, White Plains, NY 10605 USA

**Keywords:** Psychology, Diseases, Medical research, Risk factors

## Abstract

We used US nationwide commercial insurance claims data (2011–2015) to study the effect of social deprivation on clinical and demographic risk factors for suicidal ideation (SI) and suicide attempts (SA) among US youth and adults < 65 years, after having a mental health or substance use disorder-related outpatient encounter. Neighborhood social deprivation level was summarized by the quintile of social deprivation index (SDI) at individuals’ zip code level. Cox proportional hazard models were used to evaluate the effect of social deprivation on demographic and clinical risk factors for SI and SA. The study cohort consisted of 317,383 individuals < 65 years, with 124,424 aged < 25 (youth) and 192,959 aged between 25 and 64 (adults). Neighborhood social deprivation impacted risk factors for SI and SA differently for youth and adults. Among youth, SDI interacted with multiple risk factors for both SI and SA. The effects of the risk factors were larger on youth from middle socioeconomic neighborhoods. Among adults, risk of SI was the strongest in the most deprived neighborhoods, but risk of SA did not vary by neighborhood deprivation level. Our findings suggest community-based suicide prevention initiatives should be tailored according to neighborhood deprivation level and the targeted individual’s age to maximize the impact.

## Introduction

Suicide remains an important public health concern in the US^1^. Despite significant resources allocated towards suicide prevention, the rates of suicidal ideation (SI) and suicide attempts (SA) have increased over the last several years^1,2^. Numerous models have been developed to predict the risk of suicide and suicidal behaviors in clinical settings^3–5^, focusing on demographic and clinical risk factors associated with SI or SA. A recent study used electronic health records (EHR) data from multiple health systems across the US to identify important clinical risk factors for SA and suicide from over 300 potential predictors, including prior history, diagnoses, medications, healthcare service utilization, and routinely collected depression questionnaires, through developing and validating predictive models^4^. The most important risk factors identified were long-, mid-, and short-term (past 5 years, past 1 year, and past 3 months) mental health and substance use disorder (MH/SUD) related diagnosis, healthcare services utilization, and prescription medications. Using EHR data of almost 20 million visits of individuals aged 13 years or older with an outpatient mental health diagnosis, this study’s models achieved overall accuracy of prediction (c-statistic) over 80%, which significantly outperformed other published models, and thus can be considered as benchmark models for risk prediction.

As many health care systems are moving towards routinely collecting individual-level data on social determinants of health (SDoH) during a clinical encounter and integrating such information within EHRs^6,7^, it has become possible to study the effects of SDoH on health outcomes. Several studies have found associations between SDoH and suicide, including unemployment, poverty, and divorce rates^8–11^. In addition, the independent effects of SDoH on SI or SA persisted even after controlling for the demographic and clinical risk factors of individual participants. In a recent study using the EHR data from the Veterans Health Administration (VHA), adverse SDoH factors, such as housing instability, unemployment, and financial problems, were strongly associated with SI and SA even after adjusting for the presence of major depressive, alcohol use, drug use, anxiety, posttraumatic stress, schizophrenia, and bipolar disorders^12^. Further, SDoH are associated with risk factors for SI and SA. Living in disadvantaged neighborhoods has also been linked to depression^13,14^, substance use disorders in adolescents^15^, and higher utilization of health services^16,17^, all of which are risk factors for suicide and suicidal behaviors^4^. Therefore, it is important to investigate if SDoH modifies the effects of demographic and clinical risk factors for SI or SA.

Few studies have investigated the modifying effect of SDoH on demographic and clinical risk factors for SI or SA. A study based on the Canadian National Longitudinal Survey of Children and Youth data found that youth living in poor neighborhoods were at greater risk of SI and SA, and the effects of psychosocial risk factors were higher in poor neighborhoods, suggesting the need of tailoring community-based youth suicide prevention initiatives to the poverty level of the neighborhoods in order to increase their impact^18^. However, this study only considered one dimension of SDoH, neighborhood poverty, which was dichotomized and used as a binary measure. SDoH contains intangible factors from multiple dimensions, including but not limited to economics, food, education, healthcare, social supports, and environment^19^. Using poverty alone will not be able to effectively identify neighborhoods that require more health care resources. In addition, polarizing neighborhoods into “poor” and “non-poor” oversimplifies reality^20^; the majority are in the middle of the distribution^21^. To avoid false dichotomies^22^, more granular levels of SDoH should be considered in analysis.

This study utilized data on commercially insured individuals in the US. Commercial health insurance plans cover over 60% of the US population under age 65, and individuals with commercial insurance coverage tend to have higher income compared to those who have public health plan coverage or are uninsured^23^. The primary objective was to evaluate whether the risks for SI or SA vary across a wide range of neighborhood-level SDoH factors, measured by the social deprivation index (SDI) at each zip code tabulation area (ZCTA). SDI is a population-level composite measure which captures multiple dimensions of SDoH, including income, education, employment, housing, household characteristics, and transportation^24^. The independent effects of neighborhood social deprivation on SI or SA in addition to individual-level demographic and clinical risk factors were also evaluated. The second objective was to evaluate whether neighborhood social deprivation modifies the effects of clinical and demographic risk factors for SI and SA. Because youth and adults exhibit different patterns for suicidal thoughts and behaviors^1^, data on youth and on adults were analyzed separately.

## Methods

### Data source and cohorts description

This nationwide retrospective cohort study was conducted using health insurance claims data from the Health Care Cost Institute (HCCI), a non-profit, nonpartisan, independent research entity. The HCCI holds nationwide deidentified insurance claims data on beneficiaries covered by commercial or Medicare Advantage plans from four major health insurance companies in the US (i.e., Aetna, Humana, Kaiser Permanente, and UnitedHealthcare) in a manner that is compliant with the Health Insurance Portability and Accountability Act (HIPAA)^25,26^. A detailed description of the HCCI database can be found elsewhere^27^.

**Figure 1:**
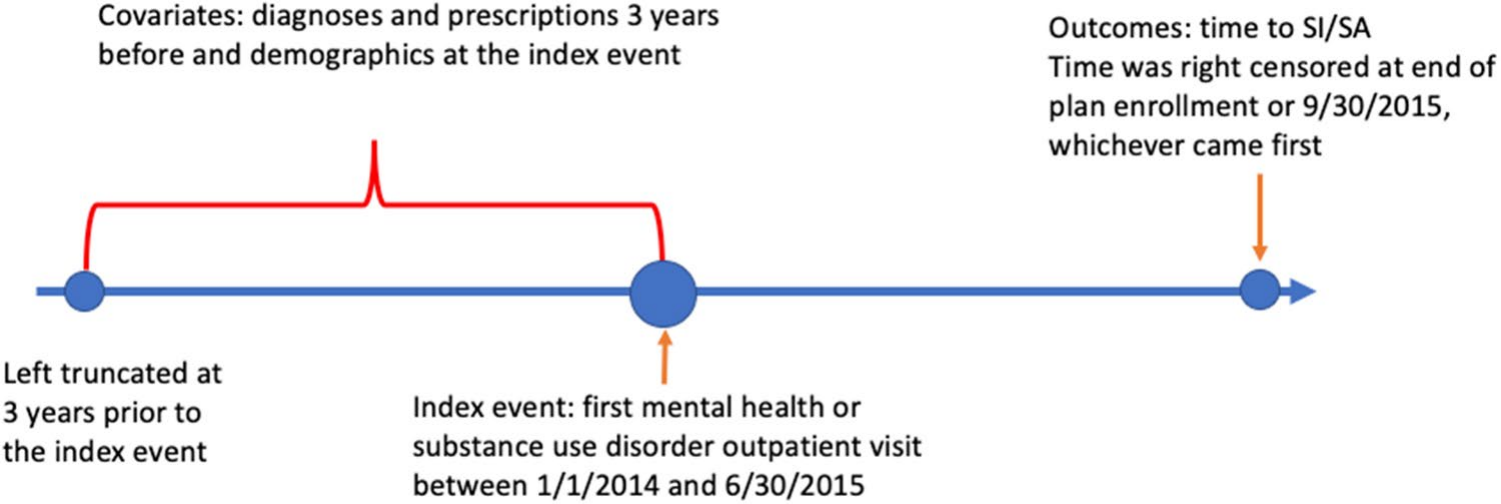
Summary of the study design.

We used the HCCI data from 2011 to 2015 to build the youth and adult study cohorts. Specifically, we restricted the cohorts to individuals who were (1) < 65 years of age; (2) subscribed to a commercial insurance plan with mental health coverage; (3) had an MH/SUD related outpatient visit between 1/1/2014 and 6/30/2015 (index event); and (4) at least 3 years of continuous enrollment before the index event. MH/SUD diagnoses were defined by the ICD-9-CM codes lists for mental health, alcohol use, and substance use summarized elsewhere^28^. For individuals with multiple index events in the study window, the first encounter was used as the index event. The three-year continuous enrollment criterion was imposed to fully capture individuals’ recent prior healthcare encounters. Figure 1 summarizes the design of the study. The data were, then, split into two cohorts: the youth and very young adults cohort included individuals younger than 25 years at the time of the index event (hereafter referred to as youth), and the other adults cohort included individuals aged 25–64 years at the time of the index event (hereafter referred to as adults).

### Independent variables

Demographic variables sex (male vs. female), age group (< 18, 18–24, 25–34, 35–44, 45–54, 55–64 years), and region (rural vs. urban) were directly extracted from the membership enrollment file in the month of the index event. Individuals’ five-digit residential zip codes were also extracted. The region (rural vs. urban) variable was derived from metropolitan statistical areas defined by the US Census core-based statistical areas (CBSAs). Individuals living in a CBSA with a population fewer than 50,000 residents were classified as living in a non-metropolitan setting.

Individuals’ neighborhood deprivation level was defined by the quintile of the social deprivation index (SDI) at individuals’ residential zip codes. SDI is a composite measure ranging from 0 to 100 developed to quantify the socio-economic variation at different area levels, i.e., counties, census tracts, Zip Code Tabulation Areas (ZCTA), and Primary Care Service Areas (PCSA). SDI combines seven socio-demographic characteristics from the American Community Survey (ACS) in a particular geographical area: percent living in poverty, percent with less than 12 years of education, percent single-parent household, percent living in rented housing unit, percent living in overcrowded housing unit, percent of households without a car, and percent non-employed adults under 65 years of age. SDI was originally developed using 2005–2009 ACS 5-year estimates and calculated at the PCSA level^24^, whereas the SDI version used in this study was updated using the 2011–2015 ACS 5-year estimates and calculated at the ZCTA level. We selected the SDI instead of other measures for two reasons: (1) social deprivation assessed with the SDI is associated with the individuals’ health and health quality^29^, as well as hospitalization and mortality risks^30^; and (2) SDI has a version developed at the ZCTA level, making it easier to map to the zip codes recorded in our claims data. Quintiles of SDI were used to allow for deprivation patterns to vary across different characteristics and to preserve the complexity of the effects of disadvantaged neighborhoods. Higher SDI quintile indicates a higher level of social deprivation.

Clinical risk factors for SI/SA included in our analysis were selected through predictive modeling in prior research^4^, which consisted of a combination of long-, mid-, and short-term diagnoses (i.e., anxiety disorders, depression, alcohol use disorders, drug use disorders, eating disorders, personality disorders, suicide attempts with schizophrenia, and suicide attempts in the past 3 years; suicide attempts in the past year; and suicide attempts in the past 3 months), medication prescriptions (i.e., antidepressant prescription filled in the past 3 months, benzodiazepine prescription filled in the past 3 months), and healthcare services (i.e., mental health inpatient stay in the past year, mental health emergency department visit in the past year, and mental health emergency department visit in the 3 months). Hospitalizations were defined by having claims in the inpatient files. ED visits were identified via claims reporting Revenue Center Codes values for emergency room services: 0450-0459 (emergency room) or 0981 (professional fees-emergency room). Diagnoses were defined by ICD-9-CM codes (details provided in Supplementary Information Table S1) and medication prescriptions were defined by national drug code (NDC) codes (The 6111 NDC codes for antidepressants and 7570 NDC codes for benzodiazepine are available upon request).

### Outcome variables

For each cohort, two outcomes were considered for survival analyses: time to SI (in days) and time to SA (in days) from the time of the index event. Survival outcomes were chosen because a binary outcome for logistic regression requires insurance beneficiaries to have continuous enrollment of the commercial health insurance plan throughout the entire period of the study, including the follow-up period post index date, which will selectively remove beneficiaries who might have changed or lost employment due to their mental health status and introduce selection bias. SI was defined by ICD-9-CM diagnosis code V62.84 and SA was defined by ICD-9-CM diagnosis codes E950-E958 and E980-E988 (undetermined intent). ICD-9-CM codes E980-E988 were included because using only E95X.X had low sensitivity in identifying a suicide attempt^31^, and including E980-E988 codes increased the sensitivity and positive predictive value of identifying a suicide attempt^4,32^. Since the US transitioned from using ICD-9-CM to ICD-10-CM codes on 10/1/2015 and our data ended on 12/31/2015, individuals were censored if they did not have an SI or SA diagnosis before 9/30/2015. We did not include SI or SA diagnoses after 9/30/2015 to avoid the potential artifacts caused by coding changes^33^. The censoring date was defined by either the last day of the month of individuals’ insurance plan enrollment or 9/30/2015, whichever came first.

### Statistical analysis

The distributions of outcome variables (time to SI and SA post index event), demographic characteristics, and risk factors were summarized for the entire cohort, each age-stratified cohort, and each age-stratified cohort by SDI quintile. Chi-square tests were used to compare the distributions of variables between the youth and the adult cohorts, as well as across SDI quintiles within each cohort.

For each outcome variable in each cohort, a sequence of Cox proportional hazards models were fit for various purposes: (1) Model 1 was fit to validate the clinical risk factors identified by others^4^, which contained all the clinical risk factors described in Section "Independent variables", (2) Model 2 was fit to evaluate the main effects of demographic variables beyond clinical risk factors, which contained clinical risk factors and demographic variables, (3) Model 3 was fit to evaluate the main effects of neighborhood-level SDI beyond clinical risk factors and demographic variables, which contained clinical risk factors, demographic variables, and SDI, and (4) Model 4 was fit to evaluate if SDI modified the effects of clinical risk factors and demographic variables, which contained clinical risk factors, demographic variables, SDI, and the interactions between SDI and clinical risk factors and demographic variables. The partial-likelihood-ratio test was used to compare the latter model with the former one, with the exception of Model 4, which was directly compared with Model 2 if the main effects of SDI were not significant (i.e., if Model 3 vs. Model 2 was not significant, then Model 4 was compared with Model 2 instead of Model 3). The latter model was selected if the partial-likelihood-ratio test was significant at 0.05 level.

All data management and analyses were performed using R statistical software (version 3.6.3; R Foundation for Statistical Computing, Vienna, Austria). All methods were carried out in accordance with the HIPAA compliance. This study was reviewed, approved, and informed consent was waived by the Institutional Review Board at Weill Cornell Medicine.

## Results

Between 1/1/2014 and 6/30/2015, we identified 317,383 individuals younger than 65 years with an index outpatient mental health visit. Among them, 124,424 aged < 25 (defined as youth) and 192,959 were aged 25–64 years (defined as adults). Youth had higher post index event prevalence rates of SI and SA than adults: The post 7-, 30-, 90-, 180-, and 365-day SI rates were 2.98%, 3.76%, 4.76%, 5.65%, and 6.66% among youth, respectively, and 1.16%, 1.50%, 1.91%, 2.36%, and 2.80% among adults, respectively. The post 7-, 30-, 90-, 180-, and 365-day SA rates were 0.40%, 0.61%, 0.92%, 1.25%, and 1.67% among youth, respectively, and 0.16%, 0.24%, 0.38%, 0.53%, and 0.69% among adults, respectively. The SI and SA rates for youths were significantly higher than those of adults at each time period of the outcome (e.g., 7, 30, 90, 180, and 365 days after the index event). Details of the distributions of the outcome variables (i.e., SI and SA rates 7-, 30-, 90-, 180-, and 365-day post index event), demographic variables, and clinical risk factors for each cohort are summarized in Table 1. Distributions of the above-mentioned variables by SDI for each cohort are summarized in Supplementary Information Table S2.

**Table 1:** Distributions of outcome variables, demographics, and risk factors by cohort.

	All N (%)	Youth N (%)	Adult N (%)	P-value (youth vs. adult)
Total	317,383 (100%)	124,424 (100%)	192,959 (100%)	
Outcome variables				
Post 7-day suicidal ideation	5967 (1.88%)	3708 (2.98%)	2238 (1.16%)	< 0.01
Post 30-day suicidal ideation	7554 (2.38%)	4678 (3.76%)	2894 (1.50%)	< 0.01
Post 90-day suicidal ideation	9617 (3.03%)	5923 (4.76%)	3686 (1.91%)	< 0.01
Post 180-day suicidal ideation	11,584 (3.65%)	7030 (5.65%)	4554 (2.36%)	< 0.01
Post 365-day suicidal ideation	13,679 (4.31%)	8287 (6.66%)	5403 (2.80%)	< 0.01
Post 7-day suicide attempt	793 (0.25%)	498 (0.40%)	309 (0.16%)	< 0.01
Post 30-day suicide attempt	1238 (0.39%)	759 (0.61%)	463 (0.24%)	< 0.01
Post 90-day suicide attempt	1873 (0.59%)	1145 (0.92%)	733 (0.38%)	< 0.01
Post 180-day suicide attempt	2571 (0.81%)	1555 (1.25%)	1023 (0.53%)	< 0.01
Post 365-day suicide attempt	3396 (1.07%)	2078 (1.67%)	1331 (0.69%)	< 0.01
Demographics				
Gender				
Male	142,121 (44.78%)	65,535 (52.67%)	76,586 (39.69%)	< 0.01
Female	175,262 (55.22%)	58,889 (47.33%)	116,373 (60.31%)	< 0.01
Age				
< 18	64,997 (20.48%)	64,997 (52.24%)		
18–24	59,427 (18.72%)	59,427 (47.76%)		
25–34	34,058 (10.73%)		34,058 (17.65%)	
35–44	48,334 (15.23%)		48,334 (25.05%)	
45–54	58,566 (18.45%)		58,566 (30.35%)	
55–64	52,001 (16.38%)		52,001 (26.95%)	
Region type				
Metropolitan	291,347 (91.80%)	116,275 (93.45%)	175,072 (90.73%)	< 0.01
Non-metropolitan	26,036 (8.20%)	8149 (6.55%)	17,887 (9.27%)	< 0.01
SDI [mean (SD)]	38.95 (27.79)	35.08 (27.37)	41.45 (27.77)	< 0.01
Clinical risk factors—diagnoses				
Anxiety disorder diagnosis in the past 3 years	137,195 (43.23%)	45,170 (36.30%)	92,025 (47.69%)	< 0.01
Depression diagnosis in the past 3 years	130,314 (41.06%)	39,447 (31.70%)	90,867 (47.09%)	< 0.01
Alcohol use disorder diagnosis in the past 3 years	30,514 (9.61%)	8652 (6.95%)	21,862 (11.33%)	< 0.01
Drug use disorder diagnosis in the past 3 years	26,142 (8.24%)	12,192 (9.80%)	13,950 (7.23%)	< 0.01
Eating disorder diagnosis in the past 3 years	6360 (2.00%)	4107 (3.30%)	2253 (1.17%)	< 0.01
Personality disorder diagnosis in the past 3 years	6279 (1.98%)	2922 (2.35%)	3357 (1.74%)	< 0.01
Suicide attempt diagnosis in the past 3 years with schizophrenia diagnosis in the past 3 years	288 (0.09%)	137 (0.11%)	151 (0.08%)	< 0.01
Suicide attempt in the past 3 years	5470 (1.72%)	2999 (2.41%)	2471 (1.28%)	< 0.01
Suicide attempt in the past year	2544 (0.80%)	1412 (1.13%)	1132 (0.59%)	< 0.01
Suicide attempt in the past 3 months	1169 (0.37%)	662 (0.53%)	507 (0.26%)	< 0.01
Clinical risk factors—medications				
Antidepressant prescription in the past 3 months	56,473 (17.79%)	14,539 (11.69%)	41,934 (21.73%)	< 0.01
Benzodiazepine prescription in the past 3 months	29,261 (9.22%)	4362 (3.51%)	24,899 (12.90%)	< 0.01
Clinical risk factors—services				
Mental health inpatient stay in the past year	19,075 (6.01%)	9195 (7.39%)	9880 (5.12%)	< 0.01
Mental health emergency department visit in the past year	18,338 (5.78%)	8521 (6.85%)	9817 (5.09%)	< 0.01
Mental health emergency department visit in the past 3 months	6079 (1.92%)	2824 (2.27%)	3255 (1.69%)	< 0.01

### Suicidal ideation in youth

Additional main effects of demographic variables to Model 1 significantly improved prediction accuracy (${\chi }^{2}(3)$=535.5, p < 0.01 for Model 2 vs. Model 1). Further addition of neighborhood level SDI to Model 2 improved prediction accuracy significantly (${\chi }^{2}(4)$=16.90, p < 0.01 for Model 3 vs. Model 2). Furthermore, Model 4, which included interactions between SDI and clinical and demographic risk factors, significantly improved the prediction accuracy of Model 3, which did not include interactions. This observation suggests that SDI significantly modified the effects of clinical risk factors and demographic variables on youth SI (${\chi }^{2}(72)$=98.55, p = 0.02 for Model 4 vs. Model 3).

**Figure 2:**
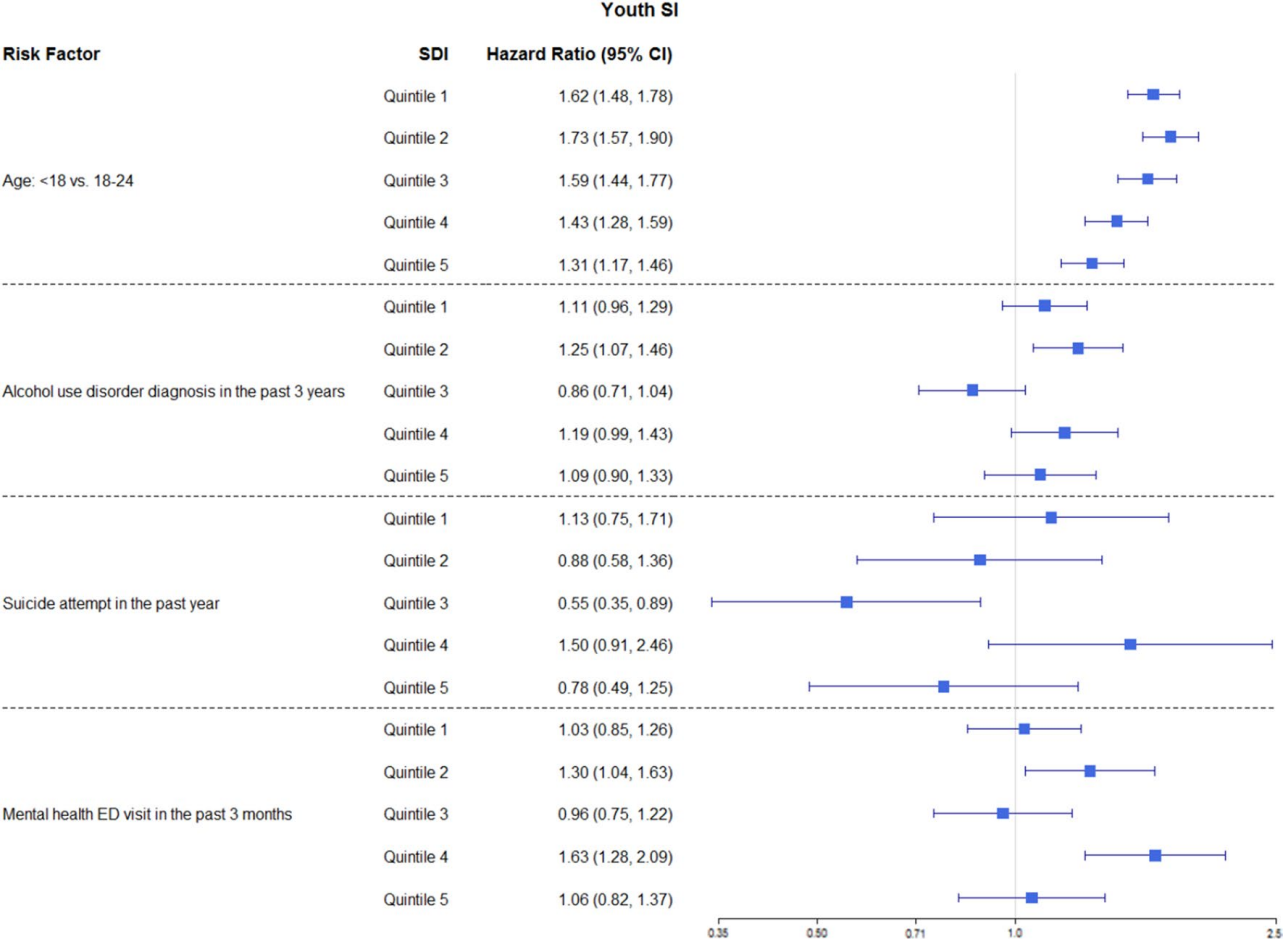
Hazard ratios of risk factors by SDI for youth suicidal ideation (SI) where the interactions were significant.

The following interactions were significant in the final model (i.e., Model 4): interactions between SDI and age, SDI and alcohol use disorder diagnosis in the past 3 years, SDI and SA in the past 1 year, and SDI and MH emergency department (ED) visits in the past 3 months (Fig. 2). Overall, the largest effect of SDI on risk factors was usually observed among middle SDI quintiles.

Specifically, youth under 18 years had higher hazard rates of SI than youth aged between 18 and 24 years; the magnitude of the elevated risk of SI decreased as the neighborhood deprivation level increased (higher SDI quintiles), with the exception of the first two quintiles, where the risks were comparable (HR = 1.62, 95% CI [1.48, 1.78]; HR = 1.73, 95% CI [1.57, 1.90]; HR = 1.59, 95% CI [1.44, 1.77]; HR = 1.43, 95% CI [1.28, 1.59]; and HR = 1.31, 95% CI [1.17, 1.46] for the 1st, 2nd, 3rd, 4th, and 5th SDI quintile, respectively). Having an alcohol use disorder diagnosis in the past 3 years was associated with an increased risk of SI for individuals from neighborhoods that belonged to the 2nd SDI quintile (HR = 1.25, 95% CI [1.07, 1.46]), but this relationship was not significant for individuals from neighborhoods of other SDI quintiles. Having mental health-related ED visits in the past 3 months was associated with an increased risk of SI for individuals from neighborhoods that belonged to the 2nd (HR = 1.30, 95% CI [1.04, 1.63]) and 4th (HR = 1.63, 95% CI [1.28, 2.09]) SDI quintiles. However, this relationship was not significant for individuals from neighborhoods of other SDI quintiles.

### Suicide attempts in youth

Additional main effects of demographic variables to Model 1 were significant (${\chi }^{2}(3)$=187.3, p < 0.01 for Model 2 vs. Model 1), but additional main effects of SDI were not significant (${\chi }^{2}(4)$=2.96, p = 0.56 for Model 3 vs. Model 2). Nevertheless, SDI still modified the effects of clinical risk factors and demographic risk factors for youth SA (${\chi }^{2}(76)$=103.8, p = 0.02 for Model 4 vs. Model 2).

**Figure 3:**
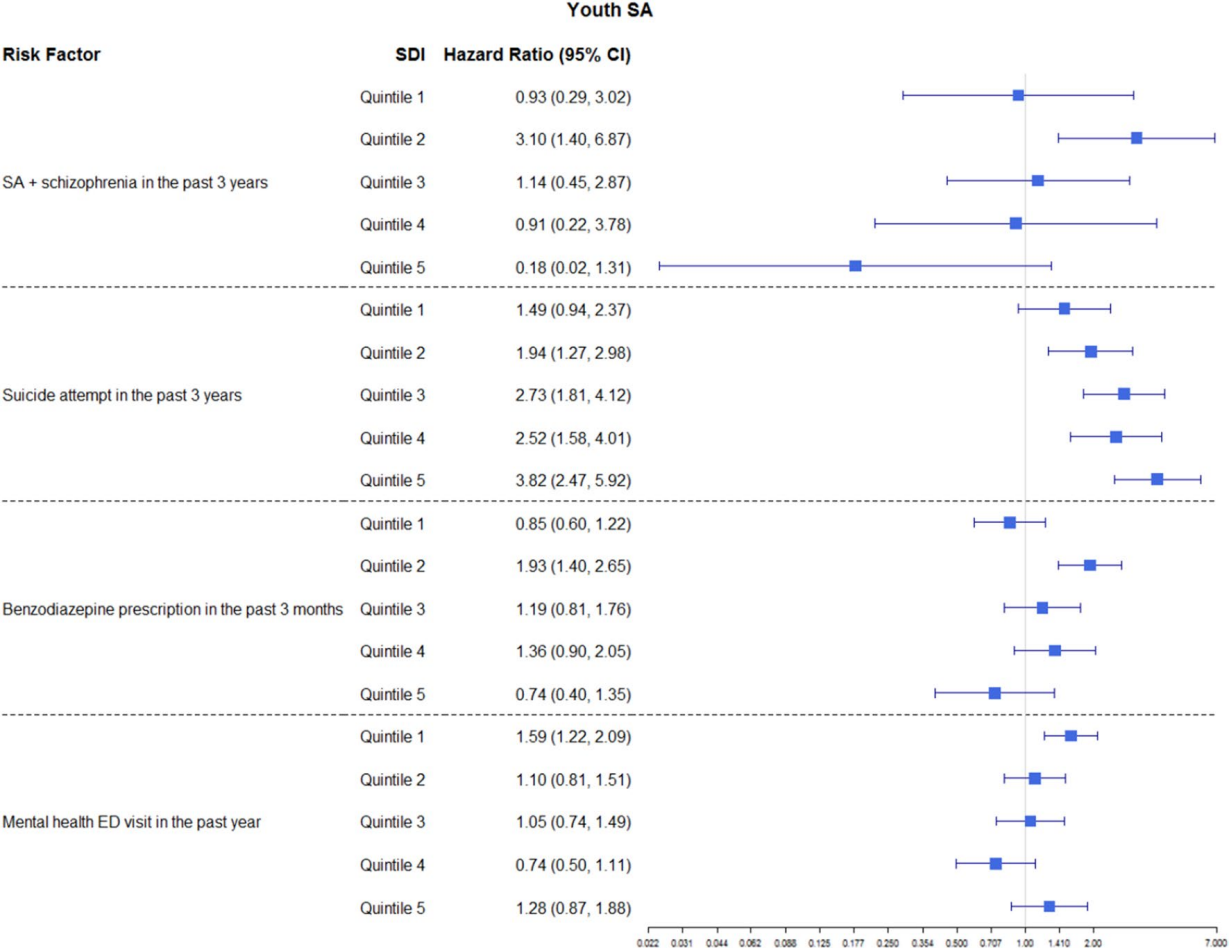
Hazard ratios of risk factors by SDI for youth suicidal attempt (SA) where the interactions were significant.

The following interactions were significant in the final model (i.e., Model 4): interactions between SDI and SA and schizophrenia in the past 3 years, SDI and SA in the past 3 years, SDI and Benzodiazepine prescription in the past 3 months, and SDI and mental health-related ED visits in the past 1 year. Hazard ratios (HR) of SA by SDI for each risk factor are summarized in Fig. 3. Similar to SI, the impact of SDI on risk factors for SA was usually the largest among middle SDI quintiles.

Specifically, having SA in the past 3 years was associated with an increased risk of SA for individuals from neighborhoods that belonged to the 2nd (HR = 1.94, 95% CI [1.27, 2.98]), 3rd (HR = 2.73, 95% CI [1.81, 4.12]), 4th (HR = 2.52, 95% CI [1.58, 4.01]), and 5th (HR = 3.82, 95% CI [2.47, 5.92]) SDI quintiles, but the association was not significant for individuals from neighborhoods that belonged to the first SDI quintile. Taken together, the effect of having SA in the past 3 years increased with neighborhood deprivation level (higher SDI quintiles). Having SA and schizophrenia in the past 3 years was associated with an increased risk of SA for individuals from neighborhoods that belonged to the 2nd SDI quintile (HR = 3.10, 95% CI [1.40, 6.87]), but this relationship was not significant for individuals from neighborhoods of other SDI quintiles. Having benzodiazepine prescriptions in the past 3 months was associated with an increased risk of SA for individuals from neighborhoods that belonged to the 2nd SDI quintile (HR = 1.93, 95% CI [1.40, 2.65]), but this relationship was not significant for individuals from neighborhoods of other SDI quintiles. Having mental health-related ED visits in the past year was associated with an increased risk of SA for individuals from neighborhoods that belonged to the 1st SDI quintile (HR = 1.59, 95% CI [1.22, 2.09]), but this relationship was not significant for individuals from neighborhoods of other SDI quintiles.

### Suicidal ideation in adults

Additional main effects of demographic variables and SDI were significant (${\chi }^{2}(5)$=154.45, p < 0.01 for Model 2 vs. Model 1; ${\chi }^{2}(4)$=31.30, p < 0.01 for Model 3 vs. Model 2). However, SDI did not modify the effects of clinical risk factors and demographic variables on adult SI (${\chi }^{2}(80)$=83.76, p = 0.36 for Model 4 vs. Model 3).

The results of the final Cox model for adult SI (i.e., Model 3) are summarized in Table 2. Specifically, SDI was positively associated with adult SI: Individuals from more disadvantaged neighborhoods had higher risk of having SI (HRs = 1.13, 1.19, and 1.25 for SDI quintiles 3rd, 4th, 5th vs. 1st; p < 0.05 for all 3 HRs).

**Table 2:** Survival analysis of suicidal ideation and suicide attempt results for commercially insured adult patients following an outpatient mental health visit.

	Adult SI HR (95% CI)	Adult SA^#^ HR (95% CI)
Demographics		
Gender		
Male	Reference	Reference
Female	0.84 (0.80, 0.89)*	0.93 (0.83, 1.03)
Age		
25–34	1.47 (1.35, 1.59)*	1.34 (1.14, 1.56)*
35–44	1.36 (1.26, 1.46)*	1.18 (1.02, 1.37)*
45–54	1.27 (1.18, 1.37)*	1.15 (1.00, 1.33)*
55–64	Reference	Reference
Region type		
Metropolitan	Reference	Reference
Non-metropolitan	0.81 (0.73, 0.90)*	1.11 (0.93, 1.33)
SDI quintiles		
Quintile 1	Reference	-
Quintile 2	1.09 (1.00, 1.19)	-
Quintile 3	1.13 (1.03, 1.23)*	-
Quintile 4	1.19 (1.09, 1.29)*	-
Quintile 5	1.25 (1.15, 1.36)*	-
Clinical risk factors—diagnoses		
Anxiety disorder diagnosis in the past 3 years	1.25 (1.18, 1.33)*	1.26 (1.12, 1.41)*
Depression diagnosis in the past 3 years	2.27 (2.12, 2.42)*	1.67 (1.48, 1.89)*
Alcohol use disorder diagnosis in the past 3 years	1.60 (1.49, 1.70)*	1.51 (1.33, 1.73)*
Drug use disorder diagnosis in the past 3 years	1.26 (1.17, 1.36)*	1.66 (1.44, 1.91)*
Eating disorder diagnosis in the past 3 years	1.28 (1.09, 1.50)*	1.44 (1.07, 1.94)*
Personality disorder diagnosis in the past 3 years	1.90 (1.72, 2.11)*	1.83 (1.50, 2.24)*
Suicide attempt diagnosis in the past 3 years with schizophrenia diagnosis in the past 3 years	1.60 (1.17, 2.18)*	1.09 (0.66, 1.79)
Suicide attempt in the past 3 years	1.51 (1.27, 1.79)*	3.29 (2.55, 4.25)*
Suicide attempt in the past year	1.05 (0.81, 1.35)	1.29 (0.90, 1.85)
Suicide attempt in the past 3 months	0.84 (0.61, 1.15)	1.20 (0.80, 1.80)
Clinical risk factors—medications		
Antidepressant prescription in the past 3 months	1.00 (0.94, 1.07)	0.99 (0.87, 1.12)
Benzodiazepine prescription in the past 3 months	1.42 (1.32, 1.52)*	1.52 (1.32, 1.75)*
Clinical risk factors—services		
Mental health inpatient stay in the past year	2.30 (2.11, 2.50)*	1.55 (1.30, 1.85)*
Mental health emergency department visit in the past year	1.42 (1.29, 1.56)*	1.29 (1.06, 1.56)*
Mental health emergency department visit in the past 3 months	1.05 (0.93, 1.18)	1.10 (0.86, 1.41)

^#^The model for SA did not have SDI because SDI was not significant when Model 3 was compared to Model 2, and therefore dropped in the final model.

*Indicates CI did not cover 1.

### Suicide attempts in adults

Additional main effects of demographic variables to Model 1 were significant (${\chi }^{2}(5)$=16.38, p = 0.01 for Model 2 vs. Model 1). However, additional main effects of SDI were not significant (${\chi }^{2}(4)$=6.66, p = 0.16 for Model 3 vs. Model 2). SDI did not modify the effects of clinical risk factors and demographic variables on adult SA (${\chi }^{2}(84)$=101.83, p = 0.09 for Model 4 vs. Model 2). Therefore, the final model for adult SA included clinical risk factors and demographic variables (i.e., Model 2).

The results of the final Cox model for adult SA (i.e., Model 2) are summarized in Table 2. For the adult cohort, having SA was not affected by the SDI level of individuals’ neighborhoods, after controlling for demographics and clinical risk factors. However, belonging to a younger age group, having anxiety disorders, depression, alcohol use disorders, drug use disorders, eating disorders, personality disorders, and suicide attempts in the past 3 years, having benzodiazepine prescriptions in the past 3 months, having mental health inpatient stay and mental health ED visits in the past year were all associated with an increased risk of SA in adults.

## Discussion

The main finding of this study is that neighborhood level social deprivation impacts the demographic and clinical risk factors of SI and SA differently among commercially insured youth and adults. Among youth, SDI interacted with many risk factors for both SI and SA. The modified effects were usually larger in individuals from less extreme SDI neighborhoods. In adults, SDI was positively associated with SI but not with SA.

Among those who had an outpatient mental health or substance use related encounter, SI and SA rates were consistently more prevalent among youth than adults. This finding aligns with a multi-center study of individuals with outpatient mental health conditions conducted during the same study period^34^. The post 90-day SA rate among youth and adults was 0.92% and 0.38%, respectively. The post 90-day SA rate among adults was within the range of post 90-day SA rates reported by Simon et al., which were 0.26% among primary care outpatient visits and 0.62% among mental health specialty visits^4^.

We found that, among youth, the impact of social deprivation on the effects of risk factors for SI and SA was usually not the largest in the most deprived or the least deprived neighborhoods. For example, having an alcohol use disorder diagnosis in the past 3 years only impacted youth’s SI among those living in neighborhoods that belonged to the 2nd SDI quintile. A potential explanation would be that parents or educators from other neighborhoods pay more attention to youth’s mental health once they are diagnosed with alcohol use disorder, so that their risk of future SI was more effectively reduced. However, more in-depth studies are needed to understand these magnified effects. Our findings on the youth cohort were consistent with an earlier study on Canadian youth where risk factors for SI and SA varied by neighborhood poverty level^18^. However, with more granular information on neighborhood disadvantage level (i.e., quintiles vs. binary), we were able to uncover that social deprivation had stronger impact on middle neighborhoods^50^, which was not apparent in the previous analysis in which a dichotomized measure of neighborhood poverty level was used.

Among adults, neighborhood social deprivation level did not modify the effects of clinical and demographic risk factors on SI or SA. However, the main effects of SDI were still significantly associated with adult SI; individuals from more deprived neighborhoods had a higher risk of having SI. The main effects of SDI were not associated with adult SA, after controlling for demographics and clinical risk factors. Several studies also reported a positive association between low income and SI^35,36^. However, one study found a positive association between relative deprivation in income and SA^35,36^, and another study observed a positive association between area deprivation and SA^37^. Among severely depressed adults, one study reported no association between area-level social deprivation and SI or SA^38^.

There are several limitations of our study. First, the deprivation level we investigated was at the individuals’ neighborhood level, which may or may not reflect the individuals’ actual socio-economic status. Second, the HCCI dataset does not provide more detailed brackets for beneficiaries under the age of 18 years. Therefore, we were unable to define the youth cohort as “aged between 12 and 25 years,” which is more commonly reported in the literature^39–41^. However, mental health problems and substance usage such as depression and exposure to drugs and alcohol are rare in very young children^42^. Therefore, we did not expect a significant portion of children under 12 in our youth cohort. Third, although fitting survival models relaxed the continuous enrollment criterion, we still imposed a three-year before index event continuous enrollment criterion in order to fully capture individuals’ prior healthcare utilization encounters. Individuals with and without continuous insurance plan enrollment might have very different socioeconomic status^27^. Fourth, since we used claims data for analysis, any encounters that were not billed to the beneficiaries' insurance plan could not be captured. Fifth, analysis in this study was conducted on the commercially insured population. Over 50% of the US population was covered by employer-sponsored health insurance plans^43^, making commercial insurance the largest insurance coverage type in the US. In our study, the average neighborhood-level percentage of population under 100% Federal Poverty Level (FPL) varied by SDI quintile, from 0.04 to 0.24 in the adults cohort and from 0.04 to 0.23 in the youth cohort (Supplementary Information Figs. S1 and S2). During the same period (2011–2015), the total US population below 100% FPL was between 14.7 and 15.9%^44^. Therefore, although the commercially insured population underrepresents people with lower incomes, in our cohorts, there was still enough variability in income to not compromise our findings. Evaluating the impact of social deprivation on risk factors for SI or SA among Medicaid enrollees will be a future direction of our study. Finally, our analysis did not include patients’ race/ethnicity information. It is well documented that suicidality, as well as risk and protective factors for suicidality vary by race and ethnicity^45–47^. A recent study also found racial/ethnic disparities in the performance of suicide predictive models^48^. Racial/ethnic minorities are disproportionately represented in deprived neighborhoods^49^, therefore, social deprivation might partially explain the racial/ethnic disparities in suicidality. Further studies are needed to evaluate whether SDI mediates the effect of race/ethnicity on suicidality.

In conclusion, our study provides information on the effects of neighborhood-level social deprivation on suicidal behaviors in youth and in adults. Community-based suicide prevention initiatives may take into considerations the targeted individuals’ age. Our findings suggest that community-based suicide prevention initiatives for adults should target severely deprived neighborhoods. Interventions for youth should focus on those residing in neighborhoods with middle social deprivation level^50^, which is the demographic group sometimes being overlooked by researchers, policy makers, and health professionals planning suicide prevention^51^.

## Supplementary Information

Supplementary Information.

## Data Availability

The data that support the findings of this study are available from Health Care Cost Institute (HCCI) but restrictions apply to the availability of these data, which were used under license for the current study, and so are not publicly available. Readers should contact HCCI for raw data.
