# Supplementary material for: Effects of social deprivation on risk factors for suicidal ideation and suicide attempts in commercially insured US youth and adults
Source: Sci Rep. 2023 Mar 13;13:4151. doi: 10.1038/s41598-023-31387-0 (PMC10011396; doi:10.1038/s41598-023-31387-0)

**Supplementary Information**

**Effects of social deprivation on risk factors for suicidal ideation and suicide attempts in commercially insured US youth and adults**

Wenna Xi, PhD^a^

Samprit Banerjee, PhD^a^

Mark Olfson, MD, MPH^b^

George S. Alexopoulos, MD^c^

Yunyu Xiao, PhD^a^

Jyotishman Pathak, PhD^a,c^

^a^Department of Population Health Sciences, Weill Cornell Medicine, New York, NY, USA 10065

^b^New York State Psychiatric Institute; Department of Psychiatry, Columbia University Irving Medical Center, New York, NY, USA 10032

^c^Department of Psychiatry, Weill Cornell Medicine, White Plains, NY 10605

Table S1. ICD-9-CM codes for alcohol use disorder, anxiety disorder, depression, drug use disorder, personality disorder, and schizophrenia diagnoses.

| Diagnoses | ICD-9-CM codes |
| --- | --- |
| Alcohol use disorder | 2910, 2911, 2912, 2913, 2914, 2915, 2918, 29181, 29182, 29189, 2919, 30300, 30301, 30302, 30390, 30391, 30392, 30500, 30501, 30502, 3575, 4255, 53530, 53531, 5710, 5711, 5712, 5713, 76071, 9800, V6542, V791, E8600 |
| Anxiety disorder | 29384, 30000, 30001, 30002, 30009, 30010, 30020, 30021, 30022, 30023, 30029, 3003, 3005, 30089, 3009, 3080, 3081, 3082, 3083, 3084, 3089, 30981, 3130, 3131, 31321, 31322, 3133, 31382, 31383 |
| Depression | 29620, 29621, 29622, 29623, 29624, 29625, 29626, 29630, 29631, 29632, 29633, 29634, 29635, 29636, 29651, 29652, 29653, 29654, 29655, 29656, 29660, 29661, 29662, 29663, 29664, 29665, 29666, 29689, 2980, 3004, 3091, 311 |
| Drug use disorder | 2920, 29211, 29212, 2922, 29281, 29282, 29283, 29284, 29285, 29289, 2929, 30400, 30401, 30402, 30410, 30411, 30412, 3042, 30420, 30421, 30422, 3043, 30430, 30431, 30432, 3044, 30440, 30441, 30442, 3045, 30450, 30451, 30452, 3046, 30460, 30461, 30462, 3047, 30470, 30471, 30472, 3048, 30480, 30481, 30482, 3049, 30490, 30491, 30492, 3052, 30520, 30521, 30522, 3053, 30530, 30531, 30532, 3054, 30540, 30541, 30542, 3055, 30550, 30551, 30552, 3056, 30561, 30562, 3057, 30570, 30571, 30572, 3058, 30580, 30581, 30582, 3059, 30591, 30592, 6483, 64830, 64831, 64832, 64833, 64834, 6555, 65550, 65551, 65553, 76072, 76073, 76075, 7795, 9650, 96500, 96501, 96502, 96509, V6542, E8500, E8501, E8502, E8541, E9350, E9351 |
| Personality disorder | 3010, 30110, 30111, 30112, 30113, 30120, 30121, 30122, 3013, 3014, 30150, 30151, 30159, 3016, 3017, 30181, 30182, 30183, 30184, 30189, 3019 |
| Schizophrenia | 29500, 29501, 29502, 29503, 29504, 29505, 29510, 29511, 29512, 29513, 29514, 29515, 29520, 29521, 29522, 29523, 29524, 29525, 29530, 29531, 29532, 29533, 29534, 29535, 29540, 29541, 29542, 29543, 29544, 29545, 29550, 29551, 29552, 29553, 29554, 29555, 29560, 29561, 29562, 29563, 29564, 29565, 29570, 29571, 29572, 29573, 29574, 29575,  29580, 29581, 29582, 29583, 29584, 29585, 29590, 29591, 29592, 29593, 29594, 29595 |

Table S2. Patient characteristics by cohorts and SDI quintiles

|  | Youth (%) | | | | | | Adult (%) | | | | | |
| --- | --- | --- | --- | --- | --- | --- | --- | --- | --- | --- | --- | --- |
|  | SDI 1^st^ Quintile | SDI 2^nd^ Quintile | SDI 3^rd^ Quintile | SDI 4^th^ Quintile | SDI 5^th^ Quintile | p (Chi-square) | SDI 1^st^ Quintile | SDI 2^nd^ Quintile | SDI 3^rd^ Quintile | SDI 4^th^ Quintile | SDI 5^th^ Quintile | p (Chi-square) |
| Total N | 31,946 | 26,893 | 23,709 | 21,293 | 20,583 |  | 33,597 | 37,340 | 38,957 | 40,474 | 42,591 |  |
| Outcome Variables | | | | | | | | | | | | |
| Post 7-day suicidal ideation | 2.76% | 3.05% | 3.13% | 2.94% | 3.13% | 0.05 | 1.05% | 1.18% | 1.14% | 1.16% | 1.27% | 0.09 |
| Post 30-day suicidal ideation | 3.43% | 3.87% | 3.98% | 3.76% | 3.85% | 0.01 | 1.36% | 1.52% | 1.47% | 1.52% | 1.59% | 0.11 |
| Post 90-day suicidal ideation | 4.53% | 4.84% | 4.96% | 4.78% | 4.74% | 0.19 | 1.76% | 1.91% | 1.90% | 1.96% | 2.00% | 0.15 |
| Post 180-day suicidal ideation | 5.41% | 5.76% | 5.90% | 5.64% | 5.62% | 0.15 | 2.18% | 2.31% | 2.36% | 2.44% | 2.45% | 0.10 |
| Post 365-day suicidal ideation | 6.48% | 6.81% | 6.78% | 6.62% | 6.62% | 0.51 | 2.58% | 2.73% | 2.83% | 2.88% | 2.94% | 0.03 |
| Post 7-day suicide attempt | 0.37% | 0.45% | 0.39% | 0.45% | 0.35% | 0.27 | 0.14% | 0.18% | 0.17% | 0.14% | 0.16% | 0.59 |
| Post 30-day suicide attempt | 0.60% | 0.70% | 0.54% | 0.64% | 0.53% | 0.11 | 0.25% | 0.25% | 0.25% | 0.21% | 0.24% | 0.80 |
| Post 90-day suicide attempt | 0.96% | 1.01% | 0.84% | 0.91% | 0.82% | 0.13 | 0.36% | 0.39% | 0.39% | 0.35% | 0.40% | 0.77 |
| Post 180-day suicide attempt | 1.29% | 1.41% | 1.21% | 1.18% | 1.11% | 0.04 | 0.53% | 0.51% | 0.55% | 0.51% | 0.56% | 0.79 |
| Post 365-day suicide attempt | 1.75% | 1.79% | 1.62% | 1.61% | 1.51% | 0.09 | 0.70% | 0.65% | 0.69% | 0.66% | 0.74% | 0.55 |
| Demographics | | | | | | | | | | | | |
| Gender |  |  |  |  |  | 0.94 |  |  |  |  |  | <0.01 |
| Male | 52.77% | 52.60% | 52.54% | 52.57% | 52.87% |  | 39.70% | 39.21% | 39.30% | 39.35% | 40.78% |  |
| Female | 47.23% | 47.40% | 47.46% | 47.43% | 47.13% |  | 60.30% | 60.79% | 60.70% | 60.65% | 59.22% |  |
| Age |  |  |  |  |  | 0.08 |  |  |  |  |  | <0.01 |
| <18 | 51.59% | 52.56% | 52.49% | 52.16% | 52.61% |  |  |  |  |  |  |  |
| 18-24 | 48.41% | 47.44% | 47.51% | 47.84% | 47.39% |  |  |  |  |  |  |  |
| 25-34 |  |  |  |  |  |  | 15.78% | 17.10% | 17.81% | 18.21% | 18.93% |  |
| 35-44 |  |  |  |  |  |  | 24.95% | 25.16% | 24.91% | 24.94% | 25.26% |  |
| 45-54 |  |  |  |  |  |  | 32.96% | 31.16% | 29.95% | 29.37% | 28.89% |  |
| 55-64 |  |  |  |  |  |  | 26.32% | 26.58% | 27.33% | 27.47% | 26.92% |  |
| Region Type |  |  |  |  |  | <0.01 |  |  |  |  |  | <0.01 |
| Metropolitan | 98.69% | 96.16% | 92.18% | 85.72% | 91.24% |  | 98.09% | 94.21% | 89.80% | 83.26% | 89.82% |  |
| Non-metropolitan | 1.31% | 3.84% | 7.82% | 14.28% | 8.76% |  | 1.91% | 5.79% | 10.20% | 16.74% | 10.18% |  |
| SDI (mean, SD) | 5.61 (3.17) | 18.43 (4.21) | 34.88 (5.33) | 55.33 (6.55) | 81.87 (9.40) |  | 5.92 (3.15) | 18.62 (4.25) | 35.10 (5.33) | 55.49 (6.57) | 81.94 (9.36) |  |
| Clinical Risk Factors – Diagnoses | | | | | | | | | | | | |
| Anxiety disorder diagnosis in the past 3 years | 39.58% | 38.25% | 36.30% | 34.64% | 30.40% | <0.01 | 48.42% | 49.19% | 48.35% | 48.10% | 44.81% | <0.01 |
| Depression diagnosis in the past 3 years | 33.18% | 33.33% | 32.10% | 30.78% | 27.79% | <0.01 | 46.93% | 48.57% | 48.05% | 47.69% | 44.47% | <0.01 |
| Alcohol use disorder diagnosis in the past 3 years | 8.04% | 7.47% | 6.88% | 6.25% | 5.42% | <0.01 | 12.10% | 11.58% | 11.49% | 10.53% | 11.12% | <0.01 |
| Drug use disorder diagnosis in the past 3 years | 10.31% | 10.38% | 9.82% | 9.34% | 8.70% | <0.01 | 7.06% | 7.20% | 7.21% | 7.19% | 7.44% | 0.34 |
| Eating disorder diagnosis in the past 3 years | 4.22% | 3.57% | 3.24% | 2.58% | 2.34% | <0.01 | 1.45% | 1.27% | 1.16% | 1.03% | 1.01% | <0.01 |
| Personality disorder diagnosis in the past 3 years | 2.47% | 2.38% | 2.29% | 2.29% | 2.25% | 0.43 | 1.73% | 1.85% | 1.87% | 1.74% | 1.52% | <0.01 |
| Suicide attempt diagnosis in the past 3 years with schizophrenia diagnosis in the past 3 years | 0.09% | 0.09% | 0.12% | 0.11% | 0.16% | 0.19 | 0.06% | 0.06% | 0.08% | 0.11% | 0.08% | 0.07 |
| Suicide attempt in the past 3 years | 2.40% | 2.42% | 2.47% | 2.35% | 2.41% | 0.95 | 1.24% | 1.27% | 1.38% | 1.24% | 1.28% | 0.40 |
| Suicide attempt in the past year | 1.16% | 1.13% | 1.19% | 1.01% | 1.16% | 0.40 | 0.61% | 0.60% | 0.62% | 0.55% | 0.56% | 0.57 |
| Suicide attempt in the past 3 months | 0.58% | 0.54% | 0.61% | 0.45% | 0.46% | 0.06 | 0.31% | 0.29% | 0.26% | 0.24% | 0.23% | 0.11 |
| Clinical Risk Factors – Medications | | | | | | | | | | | | |
| Antidepressant prescription in the past 3 months | 13.45% | 12.32% | 12.01% | 10.67% | 8.79% | <0.01 | 23.17% | 23.18% | 22.64% | 21.50% | 18.72% | <0.01 |
| Benzodiazepine prescription in the past 3 months | 3.93% | 3.63% | 3.64% | 3.22% | 2.83% | <0.01 | 13.43% | 13.27% | 13.52% | 12.84% | 11.66% | <0.01 |
| Clinical Risk Factors – Services | | | | | | | | | | | | |
| Mental health inpatient stay in the past year | 7.71% | 7.57% | 7.07% | 7.17% | 7.26% | 0.02 | 5.40% | 5.35% | 5.32% | 4.90% | 4.73% | <0.01 |
| Mental health emergency department visit in the past year | 6.85% | 6.85% | 6.65% | 6.87% | 7.05% | 0.58 | 5.10% | 5.08% | 5.18% | 5.08% | 5.02% | 0.90 |
| Mental health emergency department visit in the past 3 months | 2.46% | 2.27% | 2.04% | 2.18% | 2.33% | 0.02 | 1.89% | 1.70% | 1.83% | 1.57% | 1.50% | <0.01 |

Figure S1. Density plots of ZCTA-level percentage of population with <100% Federal Poverty Level (FPL) by the Social Deprivation Index (SDI) quintile in the youth cohort (mean = 0.04, 0.07, 0.10, 0.15, and 0.23, respectively, for SDI quintiles = 1-5).


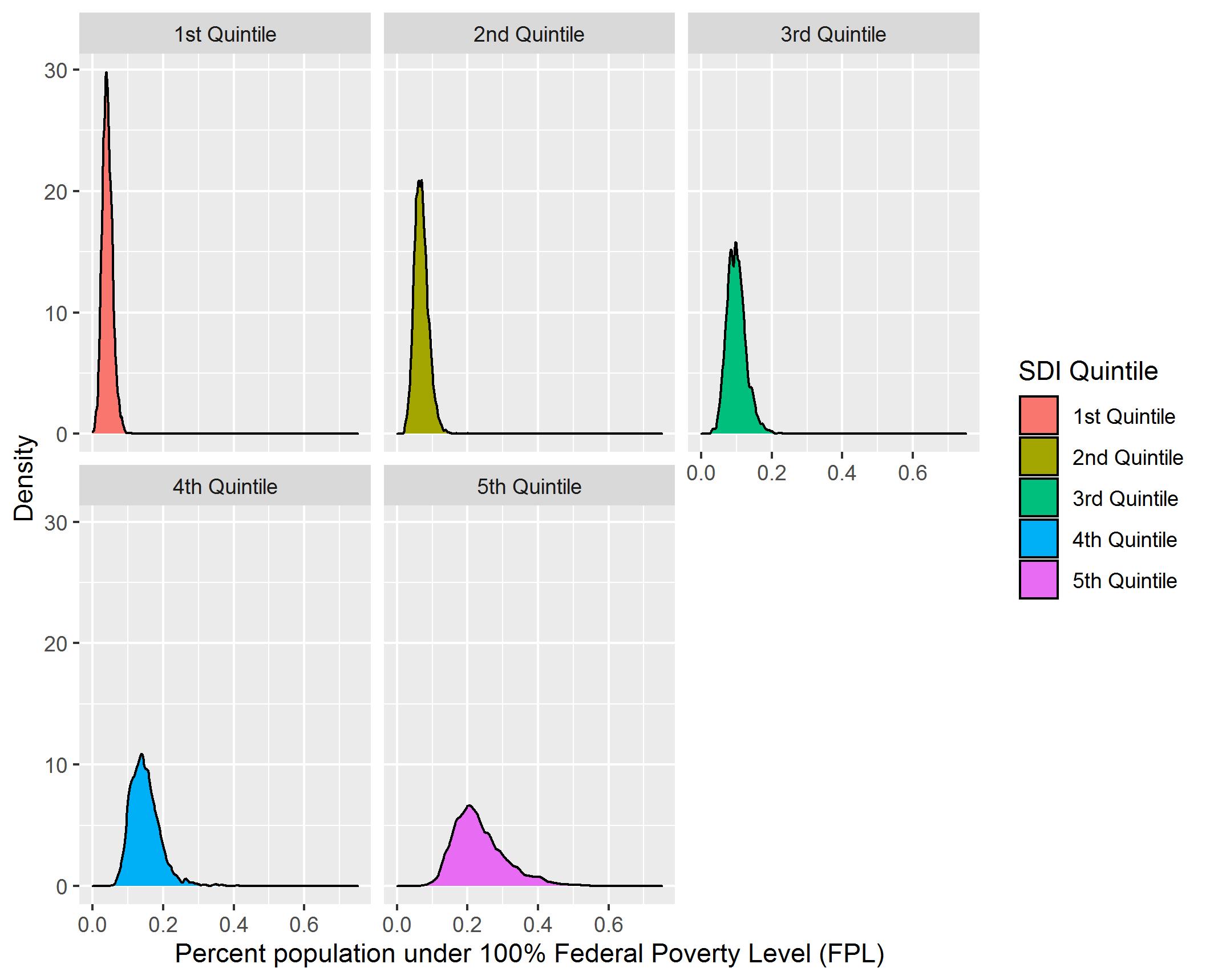


Figure S2. Density plots of ZCTA-level percentage of population with <100% Federal Poverty Level (FPL) by the Social Deprivation Index (SDI) quintile in the adults cohort (mean = 0.04, 0.07, 0.10, 0.15, and 0.24, respectively, for SDI quintiles = 1-5).


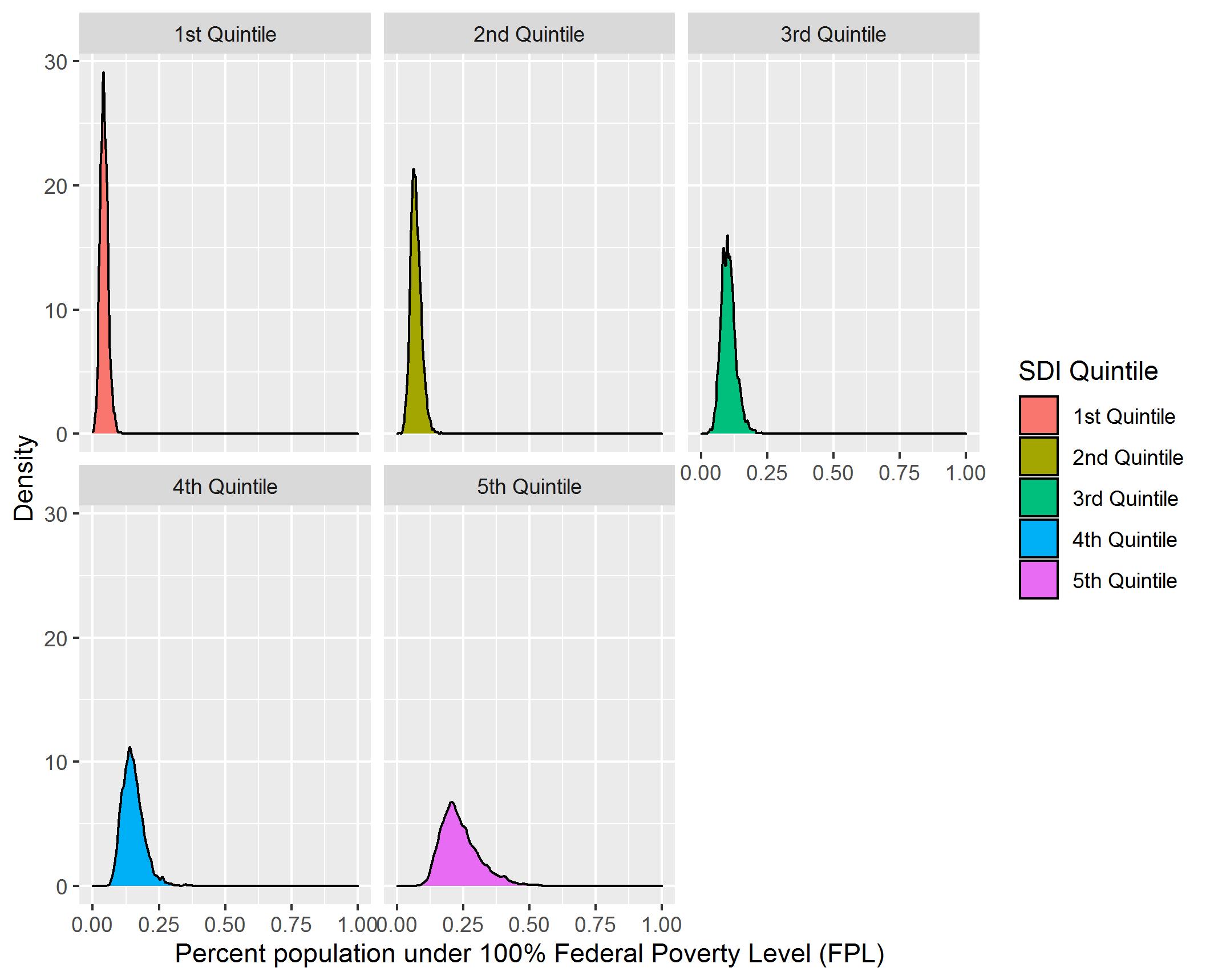

Supplement: Supplementary file 1 — Supplementary Information. [file 41598_2023_31387_MOESM1_ESM.docx]
